# Adenocarcinoma-Induced Sigmoid Colon Intussusception and Postoperative Parastomal Evisceration in an Elderly Patient: A Case Report and Literature Review

**DOI:** 10.7759/cureus.53715

**Published:** 2024-02-06

**Authors:** Jaber Alfaifi, Adeline Germain

**Affiliations:** 1 Department of General Surgery, King Khalid University, Abha, SAU; 2 Department of Hepatobiliary, Colorectal, and Digestive Surgery, University Hospital of Nancy, Nancy, FRA

**Keywords:** computed tomography, transverse colostomy complications, parastomal evisceration, colon adenocarcinoma, adult intussusception

## Abstract

Intussusception in adults is less frequent than in children, and it is less commonly seen in the colon than in the intestines. This may be explained by the fixation of the colon to the retroperitoneum. We herein describe a case of sigmoid colon intussusception caused by a sigmoid colon adenocarcinoma. An 81-year-old man presented with abdominal pain and signs and symptoms of gastrointestinal obstruction. CT revealed a “target sign” with a mass in the sigmoid colon. We diagnosed the patient with colonic obstruction secondary to intussusception of the sigmoid colon and performed an emergency transverse colostomy. On the first postoperative day, the patient had a parastomal evisceration. Oncologic resection of the sigmoid colon without reduction of the intussusception was performed. The tumor was pathologically diagnosed as well-differentiated adenocarcinoma and classified as pT3N0. The patient was discharged on the ninth postoperative day with an uneventful postoperative course. The surveillance was validated for this patient.

## Introduction

Intussusception in adults represents 1% of bowel obstruction cases and 5% of intussusceptions overall [1]. It occurs far less commonly in the colon than in the small intestine. In adults, intussusception has a different spectrum of etiologies and different principles of management. We describe a case of colo-colic intussusception in an elderly male patient caused by a malignant tumor. The initial management, including a transverse loop colostomy, was complicated by the occurrence of early parastomal evisceration, a relatively uncommon complication.

## Case presentation

**Figure 1:**
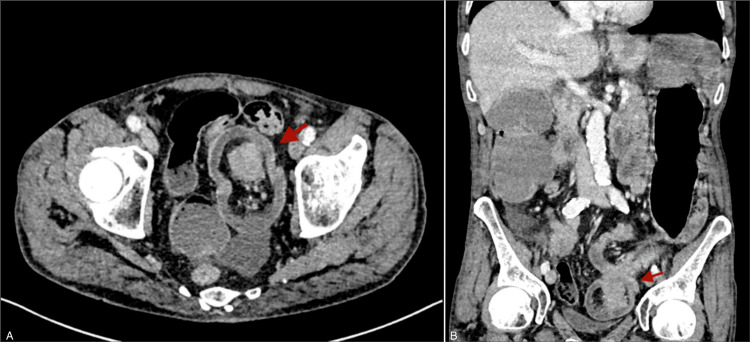
CT with intravenous contrast of the abdomen (A) Axial computed tomography (CT) scan (portal venous phase) of the abdomen shows colon wall enlargement, fat within the intussusception, and a lesion within the lumen of the colon (red arrow). (B) Coronal computed tomography (CT) scan (portal venous phase) of the abdomen shows the same finding as (A) with colo-colic intussusception.

**Figure 2:**
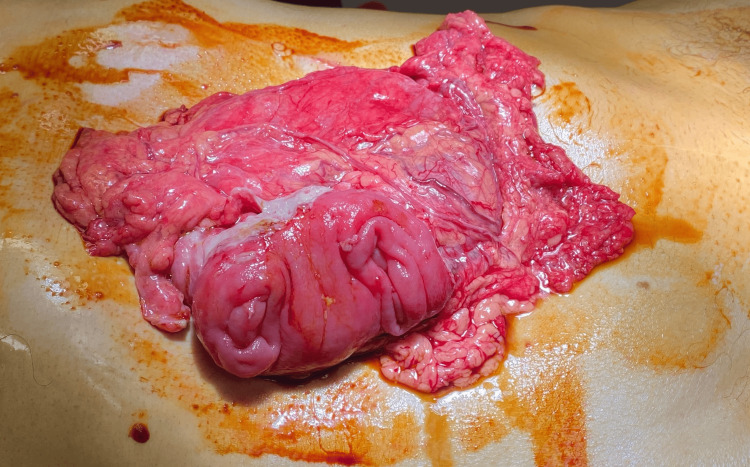
An image showing parastomal evisceration of the omentum and part of the transverse colon

**Figure 3:**
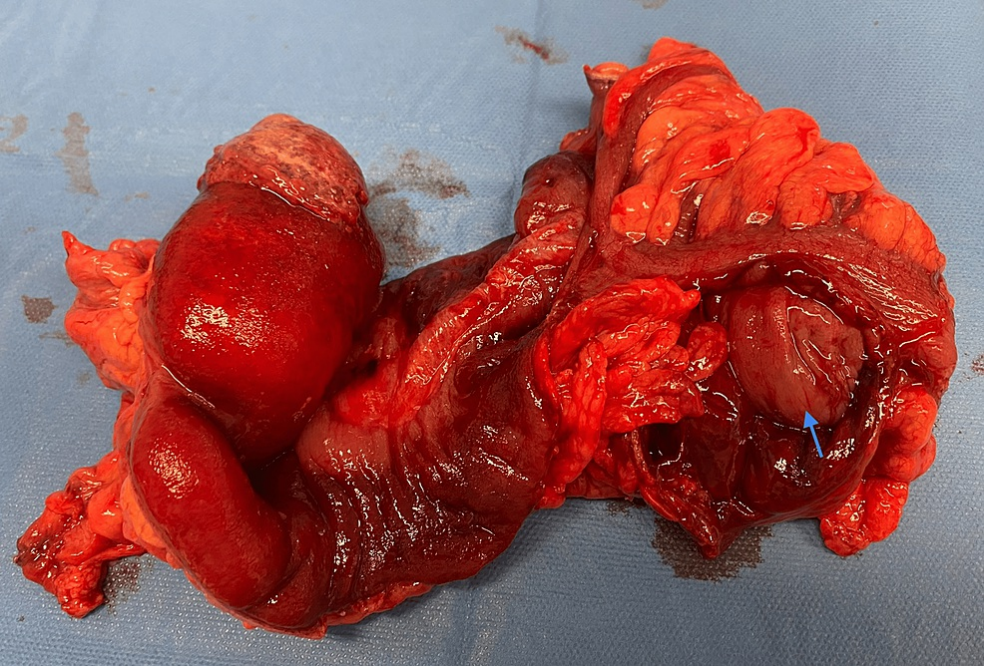
An image showing the resected part of the left colon and sigmoid colon with the colon opened to show the intussusception

An 81-year-old male patient arrived at our emergency department with abdominal pain, vomiting, and the absence of stools for 48 hours. He has a past medical history of dyslipidemia, hiatal hernia, prostatectomy for benign prostatic hyperplasia, surgery for left inguinal hernia, and subdural hematoma. A physical examination showed a distended and tympanic abdomen. The blood workup showed elevated WBC and CRP with a mild acute kidney injury. An abdominal CT scan with intravenous contrast demonstrated colon distension with a lesion of the sigmoid colon (Figure 1). The patient was operated on urgently. A transverse loop colostomy was performed by an experienced surgeon. The patient was then monitored in the ICU and had a soft stool in the colostomy bag on the first day after the operation. However, the colostomy bag had to be changed multiple times as the patient became agitated and removed it. Within a span of less than 24 hours, the patient had a parastomal evisceration of the omentum and a part of the transverse colon (Figure 2). The patient was returned to the operating room. Intraoperatively (after median laparotomy and abdominal exploration), a left colo-colic full-thickness invagination involving the sigmoid colon was identified (Figure 3). An en-bloc resection of the sigmoid colon was performed (the sigmoid colon was divided at the level of its junction with the rectum). The mechanical colorectal anastomosis was then performed. The transverse colostomy was closed by resection and anastomosis. Drains were left in the left paracolic gutter and the pouch of Douglas. The patient was monitored in the ICU for 48 hours. A return of bowel movement was observed as a soft stool on the third postoperative day. The patient was discharged on the ninth postoperative day after an uneventful postoperative course. Postoperative pathological evaluation of the resected colon revealed well-differentiated adenocarcinoma with invasion into the subserosa and negative resection margins. Thirty-one regional lymph nodes were harvested. The tumor was staged as pT3N0M0. A surveillance was validated in a multidisciplinary tumor board. The patient was seen in the clinic for a follow-up after three weeks, and he was doing well.

## Discussion

In adults, intussusception is a relatively rare condition [1,2]. This pathological phenomenon develops when a bowel segment telescopes into the lumen of the adjacent segment [3]. Typically, intussusceptions in the gastrointestinal tract occur at the junctions between fixed segments and freely mobile segments [4]. However, they may occur at any point along the small and large intestines and are categorized into four types according to their locations [5,6]. They can occur in the small intestine (entero-enteric) or between two large bowel segments (colo-colonic). The ileo-colic form of intussusception is seen when the terminal ileum invaginates into the ascending colon. The ileo-cecal valve can act as a leading point for ileo-cecal intussusception. Etiologically, intussusceptions can be benign, malignant, or idiopathic.

A pathological lead point is found in more than 90% of adult intussusceptions, with neoplasm being the most common cause [7]. Lipomas are the most common benign tumors seen in both small and large bowel intussusceptions [8]. While 30% of small bowel intussusceptions are caused by malignancies, up to 66% of large bowel intussusceptions occur due to malignant lesions [7]. Primary adenocarcinoma is the most common malignant tumor in colonic intussusception [8,9]. These lesions affect normal peristaltic bowel motility, triggering an invagination process that eventually leads to the intussusception of two bowel segments [10].

Although some studies have found that the incidence of colon intussusception decreases distally along the colon, with left and sigmoid intussusceptions occurring less frequently than right and transverse intussusceptions [11], others have reported a higher incidence of intussusception in the cecum and the sigmoid colon [12].

There has been an effort to identify predictive factors for malignancy in adults presenting with intussusception. The presence of anemia, chronic symptoms lasting longer than fourteen days, and the anatomical location of the intussusception are all independent preoperative predictors of malignancy in colon intussusception [13,14].

In this paper, we present a case of colo-colic intussusception involving the sigmoid colon caused by a lesion that acted as a leading point, manifesting as colon obstruction. The patient was operated on urgently. A transverse loop colostomy was performed by an expert surgeon to relieve the obstruction and allow for a staged procedure. Unfortunately, in less than 24 hours, the patient had a significant parastomal evisceration. This can be attributed to increased intra-abdominal pressure from a chronic cough. Due to difficulty reducing this prolapse and concerns about the patient’s stability, a second operation was performed.

Clinical manifestations of colon intussusceptions vary depending on the location and degree of intussusception. It has been found that abdominal pain, obstruction, and weight loss are the most common signs and symptoms of colon intussusception [8]. Compared to pediatric patients, bowel intussusception in adults presents less acutely, with symptoms such as rectal bleeding and palpable abdominal mass being less frequent [15]. These chronic, intermittent symptoms mimic many other gastrointestinal pathologies. Therefore, a high index of suspicion is necessary in such situations, as preoperative diagnosis may be challenging. Chand et al. reported two cases of right colon intussusception secondary to right colon cancer that were not identified on preoperative workup [16]. Both cases were identified during surgery. While right colon cancer usually presents with obstruction, these patients experienced intermittent abdominal pain and weight loss, which are not typically characteristic of cecal tumors without metastasis. The authors suggested that intermittent exacerbations of abdominal pain, especially if associated with unexplained weight loss, should raise suspicions of occult colonic cancer.

CT is considered the most precise diagnostic technique for detecting intussusception, with a diagnostic accuracy ranging from 58% to 90% [15]. It is far superior to other imaging modalities, such as ultrasound, which is largely limited by the presence of air in the distended colon or bowels in cases of obstructing intussusception [17]. The intraluminal lesion was clearly detected on the CT scan in our case. Nevertheless, it could be challenging to identify leading points on CT scans.

A colonoscopy is an option in cases of colon intussusception presenting with chronic symptoms and subacute obstruction [18]. However, in emergency situations with high suspicion of intussusception, there is no place for a colonoscopy. Due to the fragility of our patient, a colostomy was a practical and expeditious intervention to alleviate the obstruction. The colostomy was productive on the first postoperative day. Unfortunately, the patient developed early parastomal evisceration. This early postoperative complication required urgent intervention.

Abdominal stoma creation is frequently performed in general surgery for various conditions. It has a wide spectrum of complications, with incidences ranging from 10% to 82% [19]. Parastomal evisceration is a rare complication, with few cases reported in the literature. Most reported cases occur in the early postoperative period, a few days after surgery [20]. In the previously published cases, several risk factors were reported. Emergency surgery, old age, postoperative cough, chronic obstructive pulmonary disease (COPD), and elevated intra-abdominal pressure were all reported as predisposing factors in most case reports [21].

Other reported predisposing factors, such as retching, vomiting, and mechanical ventilation, are associated with increased intra-abdominal pressure. Mechanical ventilation has been identified as an independent risk factor for the development of intra-abdominal hypertension, particularly in critically ill patients and when using parameters of positive end-expiratory pressure and high tidal volumes [22,23]. Stoma prolapse and parastomal hernia are mechanical abdominal wall factors that can result in colostomy wall necrosis and eventual evisceration. In these conditions, evisceration typically manifests lately after chronic stoma prolapse and reduction [24]. Other predisposing factors have been reported, such as chemotherapy, systemic corticosteroid use, poor nutrition, alcohol abuse, and chronic smoking [21]. In our patient, three predisposing factors were present (old age, postoperative cough inducing high intra-abdominal pressure, and emergency surgery).

In the literature, three cases of transverse colostomies complicated by evisceration have been reported [24-26]. The management of these eviscerations varied from eviscerated content reduction and stoma refashioning to partial resection of the transverse colon with terminal colostomy and mucous fistula (Table 1).

**Table 1:** Summary of parastomal evisceration published cases in the literature in which transverse colostomy was performed COP: chronic obstructive pulmonary disease, AIO: acute intestinal obstruction

Author/year	Age/sex	Indication of transverse colostomy	Time of evisceration after surgery	Predisposing factors	Management
Lolis et al., 2015 [24]	48/F	Advanced rectal cancer (with ovarian and hepatic metastasis) and rectovaginal fistula	18 months	Chemotherapy, systemic corticosteroid use, postoperative cough, parastomal hernia, chronic colostomy prolapse and reduction	Eviscerated bowel reduction, colonic resection, and stoma refashioning
Salles et al., 2016 [25]	82/M	Acute obstruction secondary to adenocarcinoma of the rectum	10 days	Emergency surgery, COPD, elevated intra-abdominal pressure, mechanical ventilation, technical error	Terminal colostomy and mucous fistula after segmental resection of the transverse colon
Villa et al., 2011 [26]	69/M	Obstruction by rectal cancer	8 months	Emergency surgery, AIO, vomiting, chemotherapy, elevated intra-abdominal pressure, distal colonic lumen prolapse	Eviscerated bowel reduction and stoma refashioning

Depending on the viability of the eviscerated content, many surgical options exist, including conservative management without resection. Immediate intervention is necessary to prevent ischemia of the eviscerated content and avoid longer segmental resections. In our patient, although the eviscerated portion of the transverse colon was not necrotic, we preferred the reversal of the stoma due to the patient's condition. The patient was agitated and detached his colostomy bag many times on the first day after surgery. Additionally, the risk of evisceration would not have been mitigated with conservative measures.

In contrast to small-bowel intussusception, where reduction may be attempted before resection if there is no intestinal ischemia or suspected malignancy, it is recommended to avoid reduction and minimize surgical manipulation in cases of colon intussusception [27,28]. This can be achieved by performing en bloc resection of the involved intussuscepted segments to reduce the risk of perforation, contamination, intraluminal seeding, and tumor dissemination [6,9]. In our patient, we adhered to these principles, performing oncologic resection of the descending and sigmoid colon, including the intussuscepted segment. After colostomy closure by resection and anastomosis, a mechanical colorectal anastomosis was then performed.

## Conclusions

This case report highlights two rare conditions (sigmoid colon intussusception and parastomal evisceration) that occurred in the same patient. En-bloc resection following oncologic principles is both prudent and justified in colonic intussusception, as there is a high probability of an underlying malignancy. This report further underlines the importance of properly assessing preoperative risk factors for parastomal evisceration to reduce postoperative complications and morbidities.
